# Using Fisher information to assess stability in the performance of public transportation systems

**DOI:** 10.1098/rsos.160920

**Published:** 2017-04-26

**Authors:** Nasir Ahmad, Sybil Derrible, Heriberto Cabezas

**Affiliations:** 1 Complex and Sustainable Urban Networks (CSUN) Laboratory, University of Illinois at Chicago, Chicago, IL, USA; 2 Pazmany Peter Katolikus Egyetem, Budapest, Hungary

**Keywords:** public transportation, transit performance, Fisher information, stability, regime shift

## Abstract

Public transportation systems (PTS) are large and complex systems that consist of many modes operated by different agencies to service entire regions. Assessing their performance can therefore be difficult. In this work, we use concepts of Fisher information (FI) to analyse the stability in the performance of PTS in the 372 US urbanized areas (UZA) reported by the National Transit Database. The key advantage of FI is its ability to handle multiple variables simultaneously to provide information about overall trends of a system. It can therefore detect whether a system is stable or heading towards instability, and whether any regime shifts have occurred or are approaching. A regime shift is a fundamental change in the dynamics of the system, e.g. major and lasting change in service. Here, we first provide a brief background on FI and then compute and analyse FI for all US PTS using monthly data from 2002 to 2016; datasets include unlinked passenger trips (i.e. demand) and vehicle revenue miles (i.e. supply). We detect eight different patterns from the results. We find that most PTS are seeking stability, although some PTS have gone through regime shifts. We also observe that several PTS have consistently decreasing FI results, which is a cause for concern. FI results with detailed explanations are provided for eight major UZA.

## 1. Introduction

As cities are expanding and becoming increasingly integrated [1,2], the future of public transportation systems (PTS) is bright. In 2014, a record of 10.8 billion trips were made by public transportation in the USA [3]. Moreover, with constant urbanization and a strengthening of urban cores [4], the role that PTS will have to play in cities in the future can only increase. This is desirable for several reasons. To start, PTS can significantly help reduce traffic congestion that has a significant toll on urban economies every year. They also tend to be more sustainable than private vehicles from the viewpoint of greenhouse gas emissions [5]. Nonetheless, they are complex, and evaluating their performance over time presents significant challenges, especially as they depend on many different variables that continuously evolve over time [6]. The performances of PTS are typically evaluated by looking at a range of metrics that can vary from agency to agency but that are rarely combined to get an overall performance assessment measure of a system. For example, the Chicago Transit Authority (CTA) focuses on six core areas of service: ridership, schedule, efficiency, cleanliness, safety and courteousness [7]. In the scientific literature, many studies have developed their own performance metrics to evaluate PTS [8–13], notably wrestling with issues of scale (e.g. state versus regional versus city level) [6,8,14,15].

Recently, significant advances in data science and information science have enabled the development of new and powerful techniques to analyse urban data [16–22]. Within this general context, in this article, we introduce Fisher information (FI) and show how it can be used to combine relevant metrics into one performance measure. FI can specifically be used to measure the ‘stability’ of a system. To compute FI, we use a Python script developed by the authors and published in [23]. On the one hand, PTS consist of many modes, from bus to heavy rail, that depend on many factors. On the other hand, seasonal fluctuations in ridership are common in almost all transit systems, making the analysis of monthly data difficult.

In information theory, complex systems are considered to be dynamic, orderly and well organized, but they also have the potential to undergo abrupt changes that can dramatically alter their performance. These changes are commonly referred to as regime shifts, e.g. eutrophication of lakes and coastal oceans and regional climate change [24]. Regime shifts happen in PTS as well, from the introduction of a new transit mode to sudden and substantial changes in ridership. FI is a key method developed by Fisher [25] that offers a means of measuring the amount of information about an unknown parameter (e.g. performance) based on current observations, and it has been used to assess dynamic order in real and model systems [24,26–29]. Moreover, FI is particularly able to combine many variables to assess the overall performance and stability of a system.

FI can be significantly useful to transit planners for three main reasons. First, FI provides an effective measure of overall performance of a PTS, and in particular, it is able to detect early warning signs that may lead to regime shifts. Second, it provides practical information to transit planners on which other PTS is going or has gone through similar situations. Third, because FI is not sensitive to differences in scale of the input variables, it can be used to categorize PTS across an entire region or a country (e.g. overall assessment of transit across the USA).

The main objective of this study is to measure the stability, order and regime shifts in PTS over time in all urbanized areas (UZAs) of the USA. More specifically, this article aims to:

— recall concepts of FI and explain how it can be applied to study the stability of PTS,
— compute FI for all PTS in the 372 UZAs reported by the National Transit Database (NTD),
— analyse and interpret results in FI to detect patterns in the evolution of PTS, and
— categorize PTS based on the interpretation from the computed FI.

To achieve these goals, monthly public transit data from 2002 to 2016 were collected from the NTD [30]. In particular, we use unlinked passenger trips (UPTs) and vehicle revenue miles (VRM) for all modes reported in the NTD. Using these data, we can then compute FI for all US transit systems, and we can assess the overall pattern in the 14-year period. Overall, we find the presence of eight different patterns. Specifically, we detect regime shifts in 254 PTS, and we observe decreasing FI trends for 308 PTS, which may lead to regime shifts [31]. Additionally, we find increasing FI trends for 136 PTS. An open source Python library coded by the authors has been used to compute the FI. The code can be freely downloaded from GitHub, see [32], and a tutorial is available from the authors' main website [33]. Full details on FI are not provided here, but the reader is referred to the report of the US Environmental Protection Agency [34] and Ahmad *et al*. [23] that clearly explain how FI is calculated step by step.

## 2. Background on Fisher information

### 2.1. Fisher information

FI was developed by the statistician Fisher [25], and it offers a means to measure indeterminacy. In other words, FI can measure the amount of information about an unknown parameter *θ* that is present in observable data *X*. In this section, we briefly recall the fundamentals of FI, but more details can be found in [23,32]. Mathematically, the FI available about *θ*, *I*(*θ*), is defined as [24]

2.1 $$I(\theta )=\int {\frac{\text{d}X}{p_{0}(X|\theta )}\left[\frac{\partial p_{0}(X|\theta )}{\partial \theta }\right]}^{2},$$

where *p*_0_ (*X*|*θ*) is the probability of observing a particular value of *X* in the presence of *θ*.

In practice, it is essentially impossible to use equation (2.1) because the computation of the partial derivative (*∂p*_0_(*X*|*θ*)/*∂θ*) is required for this process, and it depends on the numeric value of the unknown parameter *θ*. Through numerous derivation steps, Mayer *et al*. [29] adapted this equation for application to real systems:

2.2 $$I=\int {\frac{\text{d}s}{p(s)}\left[\frac{\text{d}p(s)}{\text{d}s}\right]}^{2}.$$

Based on the probability of observing various states of the system *p*(*s*), equation (2.2) is the foundational form of FI used in this work. Karunanithi *et al*. [24] further simplified this equation to compute FI numerically for systems characterized by discrete data

2.3 $$\text{FI}\approx 4\sum_{i-1}^{m}{\left[q_{i}-q_{i+1}\right]}^{2},$$

where *m* is the number of states of the system. A state is defined as a condition of the system determined by specifying a value for each of the variables that characterize its behaviour [24] and *q* ≡* p*^1/2^ is the amplitude of *p*(*s*). Complete details on FI, related derivations and calculation methodology can be found in [23,34].

### 2.2. Interpretation of Fisher information

The Sustainable Regimes Hypothesis was developed to provide a construct for interpreting FI [24,26,35], and it includes four main principles that form the foundation of our interpretation of the FI results:

— A steady increase in FI indicates that the system is becoming more organized/stable.
— A system is considered to be in an orderly dynamic regime when a non-zero FI remains nearly constant over time (i.e. d⟨FI⟩/dt≈0).
— A steady decrease in FI insinuates that the system is losing its functionality, stability and the dynamic behaviour patterns are breaking down. This declining trend may provide warning of an impending regime shift [31].
— A regime shift between two stable dynamic regimes is characterized by a sharp drop in FI followed by a recovery or rebound.

As a limit of FI, we note that while it can measure whether a system is stable, orderly or going through a regime shift, it cannot discern the particular variable in *X* that is causing a particular change.

## 3. Application to public transportation systems

PTS are commonly considered as the most sustainable motorized transportation systems, and they have been present in the USA for more than a century. With the significant change in technology, economy and socio-political environment, PTS have also changed substantially over time and have undergone several regime shifts. For a history of PTS, the reader is referred to Vuchic [36]. By looking at the historical data, FI can therefore be used to track the changes experienced by PTS over time, which can help better understand patterns in ridership for instance. More specifically, we look into the FI for all UZAs in the USA as reported by the NTD for (i) rail, (ii) bus, (iii) others (i.e. modes which are neither rail nor bus) and (iv) all (i.e. overall performance).

The NTD defines two major categories of PTS modes: rail and non-rail. Moreover, among non-rail modes, the bus is by far the most predominant. The modes in the rail category used for this analysis are commuter rail (CR), heavy rail (HR), hybrid rail (YR), light rail (LR) and monorail/automated guideway (MG). The modes in the bus category used are commuter bus (CB), bus (MB), bus rapid transit (RB) and trolleybus (TB). Finally, other modes are also present, that we refer to as ‘others’, and they include: Alaska railroad (AR), cable car (CC), inclined plane (IP), street car rail (SR), demand response (DR), demand response—taxi (DT), aerial tramway (TR), ferryboat (FB), jitney (JT), publico (PB) and vanpool (VP) [30].

In this work, PTS data for all US public transportation authorities have been collected from the NTD that reports monthly data from January 2002 for four main types of data: UPTs, VRM, vehicle revenue hours and vehicles operated in maximum service (peak vehicles). Because the three latter are heavily correlated, for our analysis, we solely use UPT and VRM. UPT is defined as the number of passengers who board public transportation vehicles, and VRM is defined as the miles that are travelled by vehicles while in revenue service [30]. Another way to consider these datasets is that UPT offers an indicator of the demand for transit, while VRM offers an indicator of the supply. Moreover, we used all data points from January 2002 to December 2016.

## 4. Methodology

First, as already mentioned, the data for each PTS in each UZA are divided into four different systems: (i) rail, (ii) bus, (iii) other and (iv) all. Second, the total UPT and VRM for all the UZAs are analysed to get an idea of overall public transit patterns in the USA. As a UZA can host several transit agencies that can have multiple transit modes, each system is defined by its total number of transit modes across all agencies. As an example, for the rail mode in the Chicago UZA, there are three different rail modes run by three different transit agencies: the CTA, Metra and the Northern Indiana Commuter Transportation District. Information for both UPT and VRM for these three modes is available. As we collect two variables for each system (UPT and VRM), the Chicago rail mode is represented by six variables (i.e. two variables per mode). Overall, the Chicago has a total of 32 transit modes, giving us 64 variables.

Subsequently, using the procedure described in [23,34], FI for all UZAs present in the NTD was computed. The window size selected to measure FI was 12 for 12 calendar months since the NTD reports monthly data. Moreover, we choose a window increment of 1. In other words, we first compute the FI for January 2002 to December 2002, we then compute the FI for February 2002 to January 2003, and so on until December 2016. We then calculate the yearly average to assess yearly performance; note that this two-step process allows us to account for seasonal variations in our calculations while outputting a yearly result.^1^

On a technical note, FI requires a ‘size of state’ defined by the size of the hyper-rectangle used [24], where any point outside of this hyper-rectangle forms a new state. In this work, we calculated the standard deviation of all windows and identified the smallest value as an indicator of the stable period. We then used Chebyshev's inequality [37] that multiplies the standard deviation by two to obtain the size of state for that particular variable. More information can be found in [23,34].

As mentioned earlier, FI can increase or decrease or remain stable over a period of time. It can also undergo a sharp decrease, suggesting the occurrence of a regime shift. In this article, we define a drop in FI of 3 or greater as a regime shift. Moreover, in order to find the presence of an increasing or decreasing trend, we applied a Mann–Kendall non-parametric test [38,39] with a confidence level of 95%. This non-parametric test yields three different outputs for the overall pattern: (i) increasing, (ii) decreasing and (iii) no detectable pattern. Moreover, we also detected how much the system is able to rebound after a regime shift or a decrease. The detection of a rebound of more than 75% of an original value is defined as ‘full rebound’, whereas a rebound in between 25 and 75% of the original value is defined as a ‘partial rebound’, and failure of rebounding to at least 25% of the original value is defined as ‘no rebound’ in this analysis. If the Mann–Kendall non-parametric test failed to detect any pattern and no regime shift is spotted, then the evolution of those FI is also classified as ‘no pattern/others’. Finally, we performed a frequency analysis to find the number of UZAs that belong to the different patterns in the evolution of the FI.

## 5. Results

**Figure 1.**
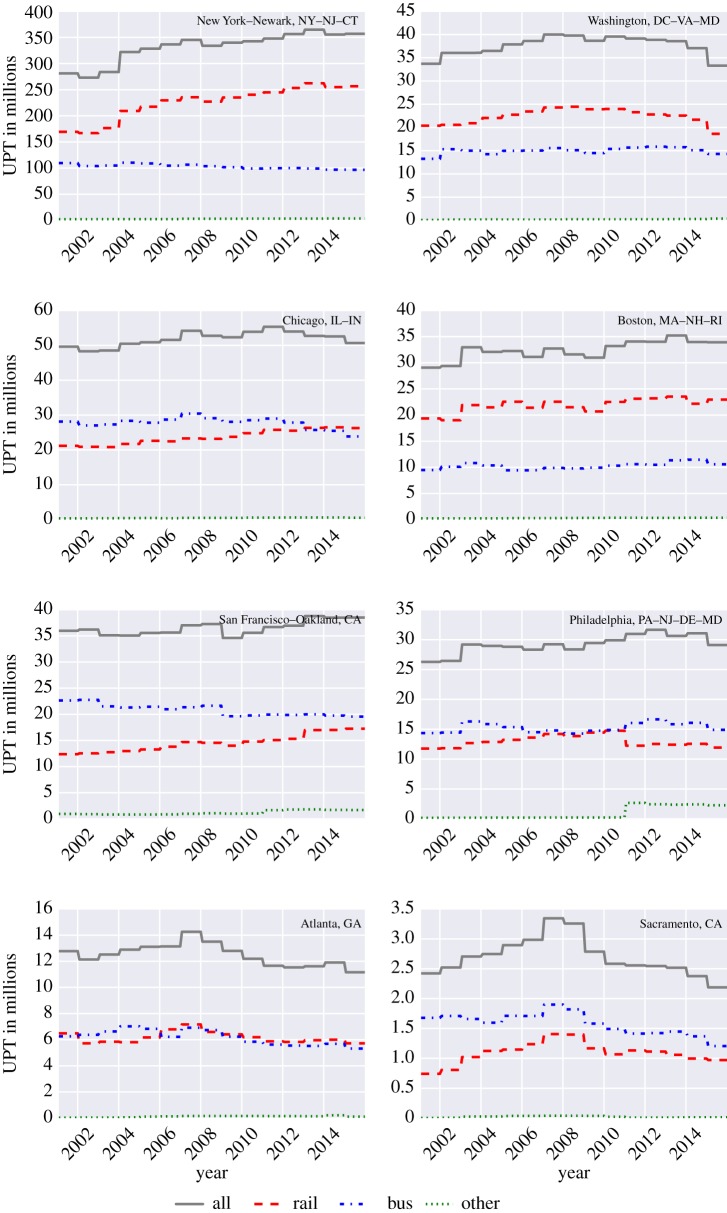
Total UPT for eight major UZAs.

**Figure 2.**
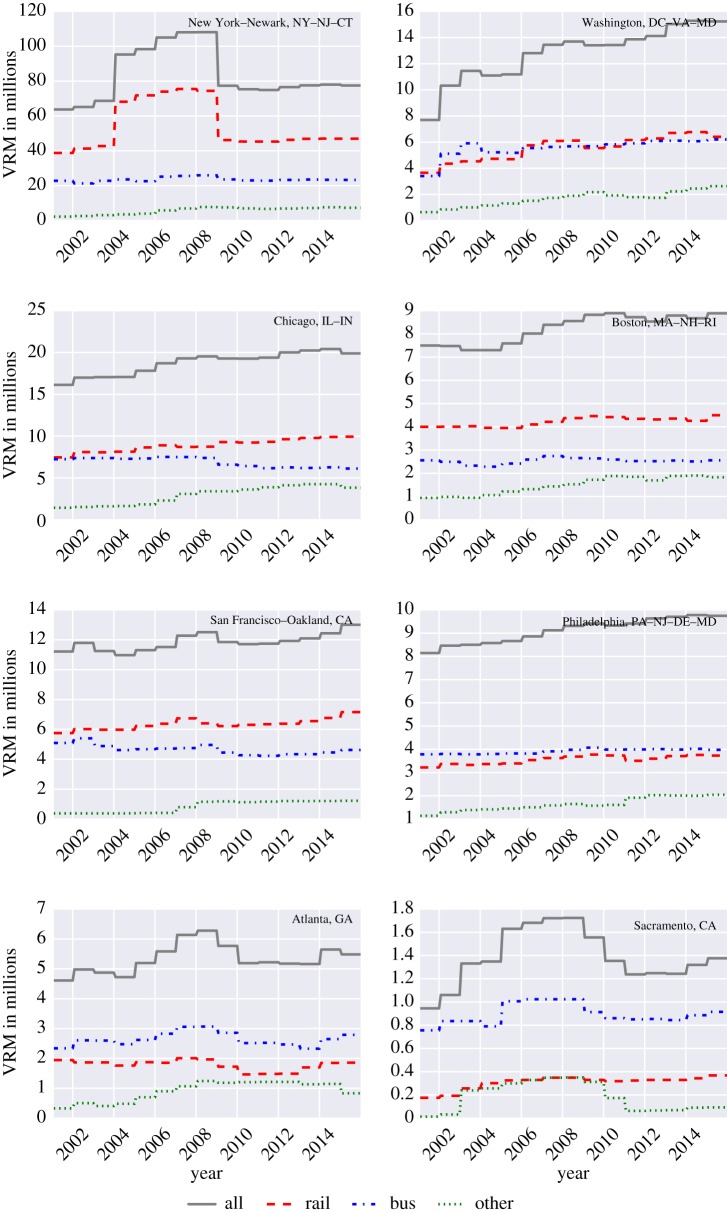
Total VRM for eight major UZAs.

The total UPT and VRM from 2002 to 2016 for eight UZAs are shown in figures 1 and 2. As defined in the NTD, the UZAs include New York–Newark, NY–NJ–CT; Washington, DC–VA–MD; Chicago, IL–IN; Boston, MA–NH–RI; San Francisco–Oakland, CA; Philadelphia, PA–NJ–DE–MD; Atlanta, GA; and Sacramento, CA. Moreover, because the data are relatively noisy, we only show yearly averages (despite the fact that we use the original monthly data for the computation of FI).

**Figure 3.**
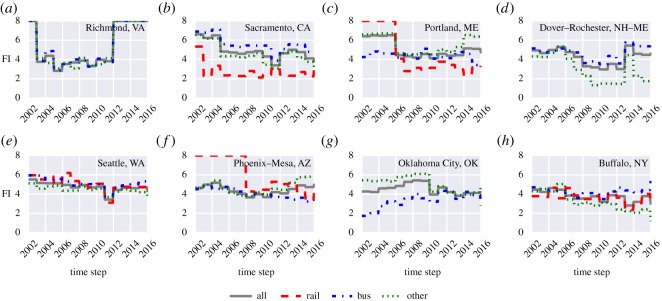
Evolution of FI for eight UZAs with: (*a*) regime shift with rebound, (*b*) regime shift with partial rebound, (*c*) regime shift without rebound, (*d*) decrease with rebound, (*e*) decrease with partial rebound, (*f*) decrease without rebound, (*g*) increase and (*h*) other.

**Figure 4.**
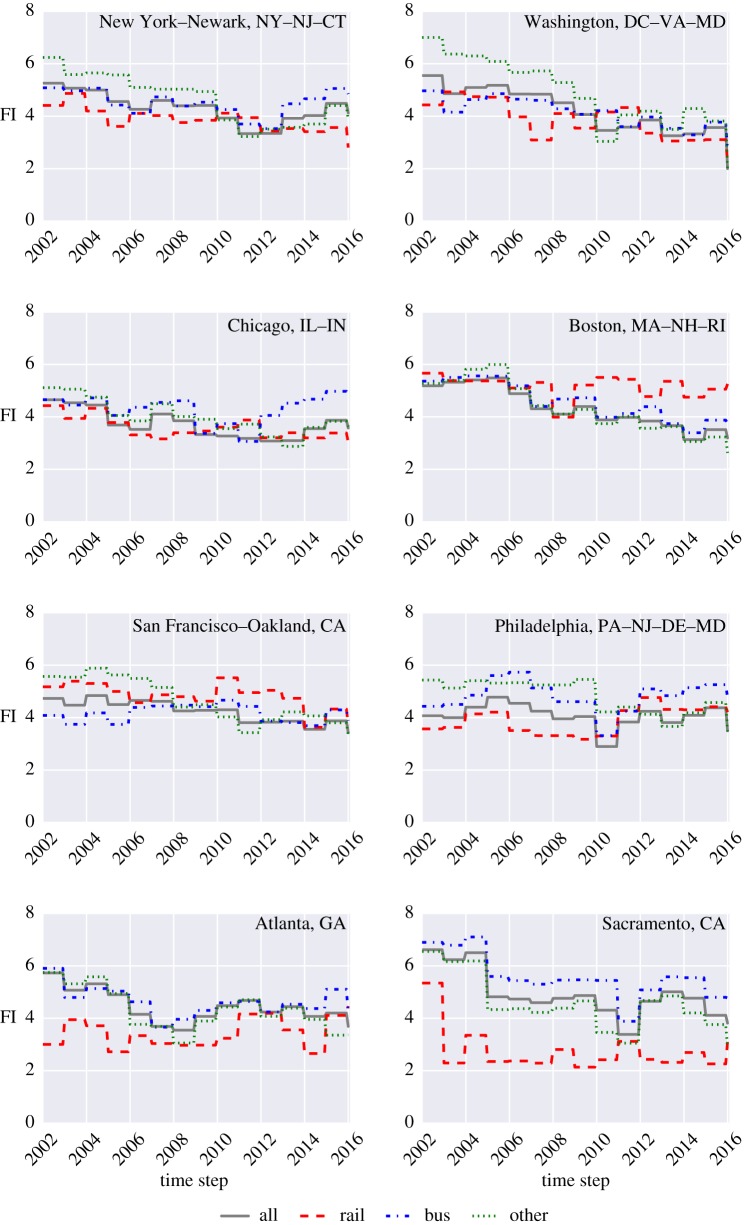
FI for eight major UZAs.

Using these data, the FI of rail, bus, other and all were calculated for all 372 UZAs reported by NTD. Overall, we observe eight different patterns in the evolution of FI based on the Sustainable Regimes Hypothesis mentioned earlier. Specifically, we are able to detect patterns for 698 out of 1146 different PTS; the other PTS either do not exist (e.g. Milwaukee, WI, does not have a rail system), or no detectable pattern can be found. The eight patterns are listed in table 1. Moreover, table 1 also shows the frequency analysis for all the four categories (i.e. bus, rail, other and all). We can notably observe 254 PTS with regime shifts. We also detect 308 PTS with decreasing FI, and 136 PTS with increasing FI. Figure 3 shows the FI for eight UZAs representing the eight different patterns observed. Moreover, the FI for the eight major UZAs used in figures 1 and 2 are shown in figure 4. The same results for all PTS can be found in [40].

**Table 1.** Patterns in the evolution of FI.

				frequency analysis			
pattern	properties	illustration	example	bus	rail	other	all
regime shift with rebound	drop in FI of 3 or greater and a rebound of 75% or greater from the minimum FI	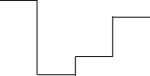	bus—Richmond, VA rail— Little Rock, AR other— Utica, NY all—Utica, NY	36	1	36	34
regime shift with partial rebound	drop in FI of 3 or greater and a rebound of 25% to 75% from the minimum FI	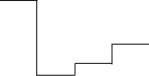	bus—Ithaca, NY rail—Sacramento, CA other—Green Bay, WI all—Corvallis, OR	25	5	24	18
regime shift without rebound	drop in FI of 3 or greater without any rebound from the minimum FI	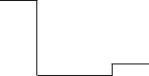	bus—Salem, OR rail—Portland, ME other— Mount Vernon, WA all—Burlington, VT	27	6	20	22
decrease with rebound	gradual decrease in FI with a rebound of 75% or greater from the minimum FI	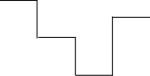	bus—Lancaster–Palmdale, CA other—Dover–Rochester, NH–ME	1	0	1	0
decrease with partial rebound	gradual decrease in FI with a rebound of 25–75% from the minimum FI	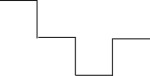	bus— Seattle, WA rail—Portland, OR–WA other—Medford, OR all—New York–Newark, NY–NJ–CT	44	8	50	59
decrease without rebound	gradual decrease in FI without any rebound	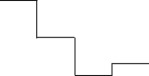	bus—Boston, MA–NH–RI rail—Chicago, IL–IN other—Eugene, OR all—Yakima, WA	40	10	38	57
increase	gradual increase in FI	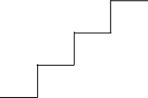	bus—New Haven, CT rail—Memphis, TN–MS–AR other—Fairbanks, AK all—Rochester, NY	45	5	45	41
no pattern/ others	no detectable pattern	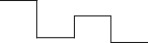	bus—Buffalo, NY rail—Springfield, MA–CT other—Portland, ME all—Raleigh, NC	154	337	158	141

## 6. Discussion

From figure 1, we can see that the total UPT for the eight major cities remained stable from 2002 to 2016, except for some minor fluctuations. In New York, the total UPT for bus and other remained flat, whereas the UPT for rail and all increased slightly in 2004 and they have continued to increase with a mild slope since 2004; this suggests that the overall changes in ridership in New York depend chiefly on the rail modes. In Washington, DC, the total UPT for all the modes remained mostly uniform throughout the period, but the ridership for rail and all followed a particularly similar pattern, suggesting a dominance of the rail mode for overall ridership patterns. The evolution of the UPT in Chicago remained stable for all four modes, and the overall ridership pattern is analogous to the pattern in the bus mode, indicating that the bus may be the dominant public transportation mode in Chicago. For Boston, and akin to Washington and New York, the rail mode is dominant. In San Francisco–Oakland, like other UZAs, the ridership patterns were stable, except for a slight decrease in the years 2003 and 2009. Moreover, we also see that bus is the major public transportation mode as it follows the trends of the overall ridership pattern. Like Chicago, in Philadelphia, the total UPT trends for all the modes were stable, and the overall pattern was similar to the bus. For Atlanta, a small increase is observed until 2007, which is followed by a gradual decrease. Furthermore, between the years 2002 and 2006, the total UPT for the bus was slightly higher than that of rail, and the ridership for the remaining years remained nearly equal. Finally, for Sacramento, the UPT for all systems increased gradually until 2009 and it then decreased steadily until 2016.

Looking at VRM, we see that except for New York, Washington, San Diego and Atlanta, the VRM of the other UZAs were stable from 2002 to 2016. New York experienced a jump in VRM in 2004, which then dropped in 2009, and remained around the 40 million mark until 2016. For Washington, DC, the VRM increased gradually from 2002 to 2016. Although rail ridership in Washington was higher than that of the bus, revenue miles for both the modes were almost similar. In Atlanta, we can observe that the VRM increased from 2004 to 2008, followed by a decrease until 2010, and then remained almost constant until 2016. Finally, the VRM patterns for Sacramento, CA, are similar to the patterns found for the UPT, except for the rail system that remained relatively stable after an increasing period between 2002 and 2005.

From figure 1, we can identify that except for New York, Washington and Boston, the bus attracts most of the riders, which is also reflected in the higher bus VRM. Nonetheless, and despite the fact that the bus is dominant in Chicago and San Francisco, the rail VRM were higher from 2002 to 2016. In Philadelphia and Atlanta, the bus VRM were higher than all other modes.

Focusing in FI results, from table 1, we can observe that the pattern ‘no pattern/others’ dominates for all categories. The majority of the UZAs for rail, bus, others and all fall in this category, which suggests that most PTS are either significantly stable or perpetually looking for stability. Among the other PTS, we can see that a significant number follow an increasing trend, including the bus systems in San Jose, New Orleans, Oklahoma City and Milwaukee. Moreover, from the PTS that experienced a regime shift, few were able to rebound completely or partially. Regime shifts are mostly observed in small UZAs because of the introduction or termination of a new mode, Sacramento offering a notable exception. In 2003, both the UPT and the VRM increase gradually, and yet the rail mode underwent a regime shift. A similar situation occurred in 2009, but no regime shift was detected. Moreover, the prime reason for rebound after a regime shift is also due to the introduction or termination of a new transit mode. In the case of a decreasing pattern (e.g. rail systems in Chicago and Washington), few PTS were able to rebound to their earlier state, and nearly an equal number of PTS have rebounded partially or failed to rebound after experiencing a decrease in FI. These results give somewhat of a cause for concern as decreasing patterns may exhibit a warning for an upcoming regime shift since uncertainty in the system is increasing (see Sustainable Regimes Hypothesis in §2.2).

Figure 3 shows examples for the eight different patterns observed overall. A regime shift with rebound was found in the bus system of Richmond, VA. The rail system of Sacramento experienced a regime shift with partial rebound, whereas the rail system in Portland, ME, also experienced a regime but it failed to rebound. A decreasing trend in FI is found in Dover–Rochester, NH–ME, as well as in Seattle, WA, and Phoenix–Mesa, AZ. However, in Dover–Rochester, NH–ME, all systems managed to rebound completely, while the Seattle, WA, transit system rebounded partially, and the Phoenix–Mesa, AZ, bus system failed to rebound altogether. Moreover, the Oklahoma City, OK, bus system shows an increasing trend in FI, and no detectable pattern was found for the PTS of Buffalo, NY.

From figure 4, we can observe that in New York, the rail, other and all modes have a decreasing trend in FI, and while the rail system was unable to rebound, the others and all categories only rebounded partially. Moreover, no detectable pattern was found for the bus system in New York. In Washington, all modes have a decreasing trend in FI, and only the other system was able to rebound partially; the other modes failed to rebound. For Chicago, no detectable patterns were found for the bus system, whereas the rail and all systems show a decreasing pattern without any rebound, and the other system shows a decreasing pattern with partial rebound. From analysing the FI for Boston, a decreasing pattern without any rebound is observed, except for the rail mode, for which no detectable patterns were found. Rail, other and all show a decreasing pattern without any rebound in San Francisco, but no detectable pattern was identified for the bus mode. For Philadelphia, a decreasing pattern with partial rebound is observed for the others and no detectable pattern was found for the remaining three systems. No detectable patterns were found for the rail and bus systems in Atlanta, whereas decreasing trends with partial rebound are observed for the remaining two. Finally, for Sacramento, a regime shift was found for the rail system in 2003, essentially capturing the increasing period for both the UPT and the VRM of the rail system.

Overall, this analysis showed no negative regime shifts; we mostly observed regime shifts due to the introduction of a new mode. While this is desirable, we also noticed that several modes seem to be on a decreasing trend, which can lead to a regime shift or dysfunction if nothing is done (e.g. bus system in Boston and rail in Chicago). An analysis of the trend in FI can therefore help transit agencies identify whether their systems are currently maintaining or losing stability, leading to possible measures to improve performance.

## 7. Conclusion

The main objective of this paper was to measure the stability, order and regime shifts of the PTS of all US UZAs by using concepts of FI. To achieve this goal, a Python code [32] was used to compute FI for all the PTS. In particular, UPT and VRM datasets from the NTD were collected and used for this analysis. FI for the bus, rail, other and all modes were computed for all 372 UZAs. We notably found the presence of eight different patterns, and the majority of the systems belong to the final category (i.e. no pattern/others). This suggests that most PTS are searching for a stable state. Among the remaining PTS, a significant number experienced a decrease in FI (i.e. 308 PTS), which is a cause for concern. By contrast, we also find optimistic trends for 136 UZAs with an increasing FI. Furthermore, several regime shifts were detected for different UZAs. A regime shift can either be positive, for example, for the case of introducing a new service (e.g. in San Diego), but it can also be negative, for example, when a needed service is terminated. Moreover, besides providing FI for eight UZAs showcasing the eight different patterns, FI for eight major UZAs were provided. Overall, PTS offer myriads of benefits, but they are also complex by nature. Considering PTS have a bright future ensuring their success is critical. This is particularly important as the era of autonomous vehicle is rapidly approaching and will likely result in a decrease in operation costs for PTS. FI offers a means to combine multiple variables of a complex system to determine its overall stability, which can prove to be valuable in practice.

## Footnotes

1 This is akin to the problem of taking the mean of the squares versus the square of the mean.

## Data accessibility

The data were collected from the National Transit Database (NTD) for this study. The Python library to compute Fisher information can be freely obtained from GitHub at https://github.com/csunlab/fisher-information.
